# 3D Bioprinted Neural Tissues: Emerging Strategies for Regeneration and Disease Modeling

**DOI:** 10.3390/pharmaceutics17091176

**Published:** 2025-09-10

**Authors:** Taekyung Choi, Jinseok Park, Suvin Lee, Hee-Jae Jeon, Byeong Hee Kim, Hyun-Ouk Kim, Hyungseok Lee

**Affiliations:** 1 Department of Smart Health Science and Technology, Kangwon National University (KNU), Chuncheon-si 24341, Republic of Korea; chlxorud0805@kangwon.ac.kr (T.C.); man4331@kangwon.ac.kr (J.P.); binee0630@kangwon.ac.kr (S.L.); jeon22@kangwon.ac.kr (H.-J.J.); kbh@kangwon.ac.kr (B.H.K.); 2 Department of Mechanical and Biomedical Engineering, Kangwon National University (KNU), Chuncheon-si 24341, Republic of Korea; 3 Department of Bioengineering, Division of Chemical Engineering and Bioengineering, College of Art, Culture and Engineering, Kangwon National University (KNU), Chuncheon-si 24341, Republic of Korea; 4 Institute of Fermentation and Brewing, Kangwon National University (KNU), Chuncheon-si 24341, Republic of Korea

**Keywords:** 3D bioprinting, bioinks, neural tissue engineering, brain model

## Abstract

Three-dimensional (3D) bioprinting has emerged as a versatile platform in regenerative medicine, capable of replicating the structural and functional intricacies of the central and peripheral nervous systems (CNS and PNS). Beyond structural repair, it enables the construction of engineered tissues that closely recapitulate neural microenvironments. This review provides a comprehensive and critical synthesis of current bioprinting strategies for neural tissue engineering, with particular emphasis on comparing natural, synthetic, and hybrid polymer-based bioinks from mechanistic and translational perspectives. Distinctively, it highlights gradient-based modulation of Schwann cell behavior and axonal pathfinding using mechanically and chemically patterned constructs. Special attention is given to printing modalities such as extrusion, inkjet, and electrohydrodynamic jet printing, examining their respective capacities for controlling spatial organization and microenvironmental cues. Representative applications include brain development models, neurodegenerative disease platforms, and glioblastoma scaffolds with integrated functional properties. Furthermore, this review identifies key translational barriers—including host tissue integration and bioink standardization—and explores emerging directions such as artificial intelligence-guided biofabrication and organ-on-chip integration, to enhance the fidelity and therapeutic potential of neural bioprinted constructs.

## 1. Introduction

The nervous system is a highly intricate network responsible for regulating sensory processing and cognitive function. Damage or dysfunction in the central and peripheral nervous systems (CNS and PNS) can lead to significant neurological impairments [1]. The global prevalence of neurological disorders is increasing, owing to the rapidly aging population. Consequently, there is a significant increase in the incidence of neurodegenerative diseases, spinal cord injuries, and peripheral nerve injuries (PNIs) [2,3,4].

Traumatic brain injury and spinal cord damage—both affecting the CNS—pose persistent therapeutic challenges [5]. PNIs, often caused by trauma or surgical complications, also remain prevalent in clinical practice. Effective treatment remains difficult owing to limited regenerative capabilities, neural structural complexity, and insufficient treatment options. Current interventions, such as surgical nerve grafting and pharmacological therapies, often fall short of restoring full function, highlighting the need for improved and customized drug delivery systems.

To address these limitations, strategies that accurately reproduce the neural microenvironment are essential. The demand for physiologically relevant in vitro models continues to increase. Neural tissue engineering (NTE)—a multidisciplinary field that integrates biomaterials, cell therapy, and advanced manufacturing technologies—has emerged as a promising approach to meet these challenges. In particular, three-dimensional (3D) bioprinting technology has recently emerged, enabling precise control of cell distribution, spatial regulation of tissue structure, and biochemical signaling pathways. This opens new avenues for creating complex, bioinspired neural tissue structures [6].

## 2. Neural Tissue

### 2.1. Neural Tissue Engineering

The nervous system consists of specialized cells called neurons, which form an intricate network for transmitting electrical impulses and information. Through this network, the nervous system regulates various physiological functions. The nervous system is anatomically divided into the CNS and PNS. The CNS, comprising the brain, cerebellum, and spinal cord [7,8], coordinates sensory and motor functions and processes incoming neural signals. Conversely, the PNS consists of nerves that extend from the CNS. The PNS transmits sensory information to the CNS and conveys motor commands to peripheral tissues and organs.

Injury to the nervous system can disrupt neuronal signal transmission and compromise the integrity of the blood–nerve barrier (BNB), leading to significant functional impairment [9]. While the PNS retains some capacity for axonal regeneration and recovery, the CNS lacks intrinsic regenerative ability. Recovery in the CNS is limited to injuries < 1 cm in length [10]. Consequently, NTE has emerged as a multidisciplinary field, aiming at promoting neuronal regeneration and functional restoration. NTE combines several biomaterials, cell-based therapies, and advanced bioprinting technologies to reconstruct damaged neural tissue.

Compared to other tissues such as skin, cartilage, and bone, nerve tissue exhibits significantly lower regenerative capacity and greater functional complexity [11]. In particular, the CNS demonstrates limited self-repair capabilities owing to the presence of inhibitory molecules, reduced neurogenesis, and highly intricate synaptic architecture [12]. Furthermore, the precise spatial organization of neurons and glial cells, the need for electrical conductivity, and the maintenance of functional synaptic connectivity pose unique challenges. Therefore, NTE must meet design criteria distinct from those of other tissues in the body, including controlled anisotropy, targeted delivery of neurotrophic factors, and reproduction of the native electrophysiological environment.

**Figure 1:**
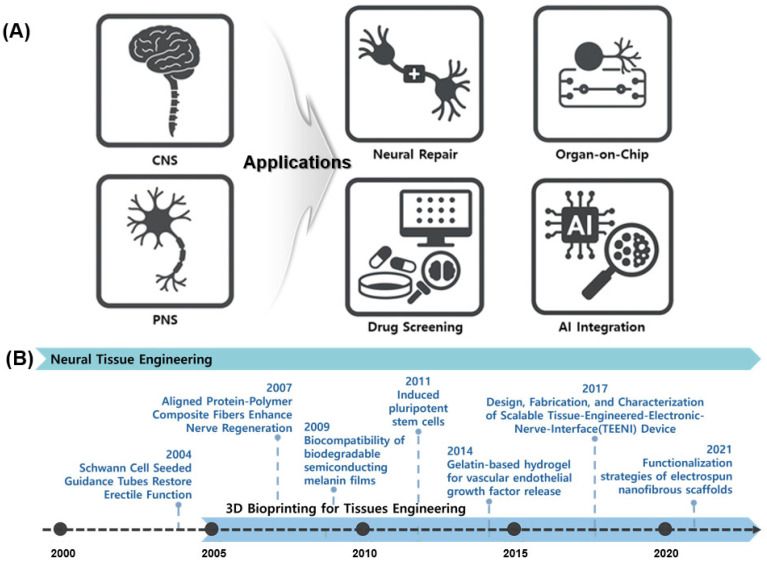
Overview of NTE and its diverse applications. (**A**) Representative applications of neural tissue engineering, highlighting its role in addressing CNS and PNS injuries through strategies such as neural repair, organ-on-chip models, drug screening platforms, and integration with AI. (**B**) Timeline of key technological advancements in NTE.

NTE has recently expanded into multiple applications (Figure 1A), with a primary focus on evaluating cerebral recovery [13]. Precision-engineered biomaterial constructs, such as Nerve Guidance Conduits (NGCs), are being developed [14]. Pathological research and drug response validation are advancing via NTE technologies and the accurate simulation of the nervous system enabled via organ-on-chip platforms. Three-dimensional bioprinted brain tissue models are utilized in drug screening owing to their superior performance compared to those of two-dimensional (2D) cell cultures and animal models [15]. The field of Artificial intelligence (AI) research is experiencing significant growth. AI integration forecasts biological responses, automates image analysis, and enhances bioprinting parameters [16].

Figure 1B shows the key advancements in NTE. This timeline highlights the progression of NTE, beginning with early strategies such as Schwann cell-induced conduits (2004), followed by the development of biodegradable semiconductor materials (2009), stem cell-based therapies (2011), and gelatin-based hydrogels for the controlled release of growth factors (2014) [17]. Recent advancements include functionalized nanofiber scaffolds introduced in 2021, which demonstrate the ongoing significant development and research activity within the field. Current technologies include Schwann cell application [18], neural conduit creation via electrospinning [19], nerve regeneration studies involving mechanical and electrical stimulation [20], and the application of stem cells and growth factors [21]. Traditional 2D cell culture methods fail to replicate the 3D complexity of brain networks [22,23]. Animal models are limited based on ethical considerations, interspecies variability, and restricted access to human neuro tissue [24,25]. To address these challenges, researchers are meticulously developing 3D brain tissue models via precise cellular organization [26,27]. NTE leverages 3D bioprinting to replicate biologically relevant structures.

### 2.2. Limitations of Conventional Fabrication Methods in Neural Tissue Engineering

In NTE, traditional fabrication methods—such as solvent casting, freeze-drying, and electrospinning—have been widely employed to produce porous scaffolds that facilitate neural cell adhesion. However, these approaches have significant limitations in replicating the intricate architecture and functional complexity of neural tissues. Solvent casting, often combined with powder extrusion, is commonly used to fabricate porous polymer scaffolds. However, this method limits precise control over pore shape and distribution, leading to uneven cell distribution and reduced nutrient transport efficiency. Residual solvents can cause cytotoxicity, rendering this approach unsuitable for highly sensitive neural tissue environments [28]. Freeze-drying is a widely effective method for producing scaffolds with high porosity and biodegradability, but the resulting structures often lack mechanical strength and fail to reproduce the unique microenvironment of neural tissue, such as axial anisotropy [29]. Electrospinning produces nanofiber-based scaffolds that mimic the architecture of the extracellular matrix (ECM). A report shows that aligned electrospun fibers are effective in inducing neural stem cell differentiation and guiding neurite outgrowth. However, most electrospinning technologies yield 2D or pseudo–3D con-structs, with limited capability to control multilayered cell arrangements or vertical cell distribution [30]. To address the limitations of existing fabrication methods, 3D bioprinting—capable of precisely depositing bioinks in digitally programmed layers—is gaining attention as a next-generation approach for effectively reproducing the complex microenvironment of neural tissue. These techniques can integrate various cell types, growth fac-tors, and anisotropic physical properties within a single construct.

### 2.3. Role of Three-Dimensional Bioprinting in Neural Tissue Engineering

**Figure 2:**
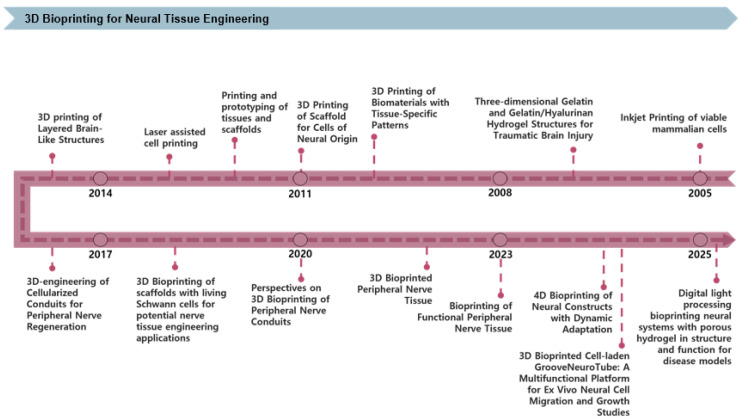
Timeline of key advancements in 3D bioprinting technologies applied to NTE. The timeline highlights significant milestones, beginning with early inkjet printing of viable mammalian cells, followed by developing biomaterial-based scaffolds and prototypes, laser-assisted cell printing, and cell-laden peripheral nerve conduits. Recent advances feature 4D bioprinting for dynamically adaptive neural constructs and DLP-based porous hydrogels for advanced neural tissue models suitable for disease studies.

3D bioprinting technology is becoming increasingly significant in NTE, enabling precise structural control, enhanced cell-scaffold interactions, and the creation of functional tissue models. Figure 2 shows the progression of 3D bioprinting technology over recent decades. Initial studies investigated glial and retinal cell viability and neurite outgrowth employing inkjet-based printing techniques [31]. Subsequent work explored traumatic brain injury therapy via GelMA/HA-based hydrogels [11], marked the first formal application of biomaterials and tissue-specific patterning in NTE. The technology was further enhanced to incorporate neural-based cell printing as well as fabricate tissue and scaffold prototypes, followed by integrating high-resolution laser printing to enhance structural precision [32,33]. Since 2014, research on fabricating nerve guidance conduits incorporating cells for peripheral nerve regeneration has garnered interest [34,35]. Scaffold development using Schwann cells has become a central focus in PNS regeneration [36]. Recent research has intensified on applying 3D bioprinting to peripheral nerve conduits and tissues, while 4D bioprinting has emerged as a strategy for creating dynamically adaptive constructs [37]. Recently, high-resolution brain cell scaffolds have been fabricated using digital light processing-based porous hydrogels [38]. Additionally, a multilayered blood–brain barrier (BBB) model has been effectively developed and applied to drug permeability investigations [39]. Consequently, 3D bioprinting technology is advancing beyond the creation of rudimentary structures towards the development of tissues with physiological function and dynamic responsiveness.

## 3. Fundamentals of Three-Dimensional Bioprinting for Neural Tissues

### 3.1. Bioprinting Technologies

3D bioprinting employs computer-aided design and manufacturing technologies to precisely deposit biomaterials at specified locations, enabling the fabrication of complex 3D biological structures [40]. Scaffolds for nerve tissue regeneration must meet various characteristics, including biocompatibility, biodegradability, appropriate porosity, and sufficient mechanical strength [41], while also providing an environment that effectively supports cell adhesion and viability. Conventional scaffold manufacturing methods—including solvent casting, particle leaching, phase separation, and freeze-drying—offer limited control over microporosity. Furthermore, residual organic solvents can impair cell survival [42]. To address these limitations, additive manufacturing technology has been adopted, enabling more precise structural design and the fabrication of complex tissues [43]. Currently, bioprinting technology is primarily divided into four main categories: extrusion-based, inkjet-based, electrohydrodynamic (EHD) and laser-assisted. Each method operates on distinct principles and presents specific advantages and limitations [44].

As summarized in Table 1, each bioprinting technology presents distinct advantages and disadvantages from both mechanistic and translational perspectives. Extrusion-based bioprinting allows the use of bioinks with diverse viscosities, making it suitable for scalable and clinically relevant constructs; however, the application of high pressure can generate excessive shear stress that compromises cell viability. Inkjet-based bioprinting enables the fabrication of high-resolution structures under biocompatible printing conditions, but its applicability is restricted to low-viscosity bioinks, which limits scalability for larger tissue constructs. Laser-assisted bioprinting provides excellent precision and patterning capabilities, but it is costly, and cell viability can be affected by the duration of laser exposure. EHD bioprinting offers nanoscale resolution and fine droplet control, providing opportunities for fabricating complex tissues, but it suffers from low throughput, high voltage risks, and complex setup requirements.

#### 3.1.1. Extrusion-Based Bioprinting

**Figure 3:**
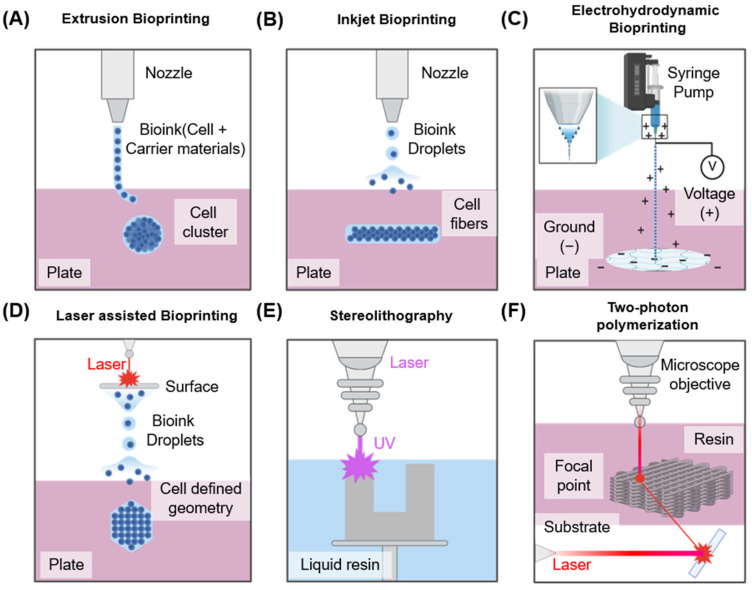
**Overview of bioprinting technologies used in tissue engineering, illustrating five different bioprinting methods:** (**A**) Extrusion-based bioprinting using mechanical force for cylindrical construct formation. (**B**) Inkjet bioprinting employing piezoelectric actuators for droplet deposition, (**C**) EHD bioprinting utilizing electric fields to generate ultra-fine jets or droplets for high-resolution bioink patterning. (**D**) Laser-assisted bioprinting involving laser-induced droplet ejection for precision cell placement. (**E**) Stereolithography utilizing patterned light projections to fabricate scaffolds with intricate structures. (**F**) Two-photon polymerization employing a femtosecond laser for highly precise, vascularized tissue models.

Figure 3A shows an extrusion-based bioprinting method. In this approach, bioink is extruded via a nozzle to fabricate a desired structure. Pneumatic or mechanical systems—including screw- or piston-driven mechanisms—are typically utilized to control extrusion. This technique accommodates various bioink viscosities, and single- or multi-nozzle configurations can be employed to extrude multiple inks simultaneously [45,46]. Nevertheless, this technique generally offers relatively low printing resolution [47], and the shear stress generated during extrusion can adversely affect cell viability [48]. Despite these limitations, it remains technically simple and cost-effective, making it the most widely adopted bioprinting technique to date. With careful nozzle control and optimized bioink formulation, it can achieve sufficient reproducible results even in complex structures such as neural tissues.

#### 3.1.2. Inkjet-Based Bioprinting

The inkjet-based bioprinting method (Figure 3B) creates structures via ejecting discrete droplets of bioink onto defined positions. It is best suited for bioinks with low viscosity [49,50]. Two principal droplet-generation mechanisms are employed. The primary technique is thermal inkjet, which applies heating to produce vapor bubbles within the bioink, thereby expelling droplets. The alternate technique is the piezoelectric inkjet method, which utilizes piezoelectric actuators to eject bioink via voltage stimulation. This technique generally offers higher cell viability and enables the fabrication of high-resolution structures. However, nozzle clogging occurs with high-viscosity bioinks, limiting the range of suitable inks [49]. While well-suited for neural tissue printing requiring precise positional control, it remains constrained owing to the limited mechanical strength of the resulting constructs.

#### 3.1.3. Electrohydrodynamic (EHD) Bioprinting

Electrohydrodynamic (EHD) bioprinting is an emerging high-resolution printing technology that uses electrostatic forces to eject ultra-fine bioink droplets from a nozzle (Figure 3C). Unlike conventional extrusion-based bioprinting that depends on mechanical pressure to extrude ink, EHD bioprinting applies a high-voltage electric field (typically 0.1–10 kV) between the nozzle and the substrate [51]. This electric field forms a Taylor cone at the nozzle tip, from which charged droplets are ejected, enabling nanoscale patterning with exceptional spatial resolution and precision in scaffold fabrication [52]. One of the primary advantages of EHD bioprinting is its ability to process low-viscosity bioinks with minimal mechanical shear stress, thereby supporting high cell viability. However, the process is highly sensitive to various variables such as voltage, nozzle–substrate distance, flow rate, ink conductivity, and viscosity. High voltage can induce electrical stress on encapsulated cells, potentially compromising their survival and function [53]. Maintaining sterility and structural uniformity across large constructs also remains a significant challenge.

#### 3.1.4. Laser-Assisted Bioprinting

Laser-based bioprinting is a nozzle-free, non-contact technique capable of producing high-resolution structures (Figure 3C). In this method, a pulsed laser is used to apply heat to a thin metal film. The resulting rapid vaporization generates a pressure wave that propels the biological material onto the substrate, forming the desired structure [54]. This approach accommodates bioinks of various viscosities and facilitates the fabrication of fine structures, which is advantageous for controlling cell alignment and spatial distribution. However, cells may be damaged during the laser irradiation process owing to heat or emitted residual materials, potentially affecting their viability [55,56]. Therefore, when handling sensitive nerve cells, precise control of laser intensity and pulse duration is essential. Additionally, Figure 3D,E shows the various bioprinting techniques utilized [57,58].

### 3.2. Bioinks for Neural Tissue Engineering

Post-stacking shape and structural stability largely depend on the rheological properties of bioink. Key parameters include shear-thinning behavior, viscoelasticity, and viscosity [59]. Most bioinks are polymer-based, including natural, synthetic, composite, and hybrid types. Shear-thinning is essential for minimizing cell damage. It reduces printing viscosity to enhance ink flow, then increases viscosity to strengthen the construct [60]. Viscoelasticity governs the balance between ink flow and structural integrity [61]. Generally, a higher storage modulus than that of the loss modulus usually enhances structural stability. However, excessively high values can clog nozzles or cause irregular printing [62]. Bioink properties significantly influence cellular activity. Advances in formulation are yielding bioinks optimized for specific applications, incorporating natural polymers, synthetic polymers, and nanoparticles [63].

As summarized in Table 2, various types of bioinks exhibit distinct properties from mechanical and translational perspectives. Natural polymers such as collagen, alginate, and dECM provide excellent biocompatibility that supports neural survival and differentiation, but their limited mechanical strength, rapid degradation, and variability restrict long-term stability. Synthetic polymers such as PEG, PCL, and PLA allow precise control over mechanical and degradation properties and provide high reproducibility, but they lack the biological cues necessary for neural tissue development. Hybrid polymers aim to combine the advantages of natural and synthetic systems by integrating bioactivity with mechanical robustness, and more recently, conductive polymers such as PEDOT:PSS have been incorporated to provide electrical functionality for neural stimulation; however, issues related to cytotoxicity, degradation byproducts, and manufacturing complexity must be resolved before clinical application.

#### 3.2.1. Natural Polymers

Natural polymers were the first materials used in neural tissue engineering. Derived from biological sources, these polymers demonstrate physicochemical and structural properties similar to those of native tissues. They exhibit excellent high biocompatibility and biodegradability. Collagen-based hydrogels, a representative example, enhance neural survival and proliferation and are primarily used as neural induction conduits [64,65]. Hyaluronic acid (HA), a key component of the extracellular matrix, promotes nerve outgrowth and cell differentiation, making it valuable in treatments for both PNS and CNS [66]. Chitosan-based hydrogels enhance cell adhesion and nerve outgrowth, being primarily applied in PNS and CNS regeneration [67]. Alginic acid, a natural polysaccharide derived from brown algae and certain bacteria, is effectively used in peripheral nerve regeneration [68].

Recently, decellularized extracellular matrix (dECM)-based bioinks have attracted increasing attention. dECM preserves the ECM components and bioactive factors of the source tissue, facilitating cell-specific adhesion and functional differentiation while providing a tissue-specific biochemical and structural environment [69]. dECM derived from the PNS or CNS promotes maturation of neurons, Schwann cells, and synapse formation [70]. When combined with bioprinting technology, dECM is increasingly applied to customized neural tissue modeling and regenerative therapy platforms. While natural polymers exhibit high biocompatibility and promote neural cell growth, they often lack sufficient mechanical strength and structural stability. For example, both collagen and HA support cell proliferation but degrade rapidly, compromising long-term structural integrity. Chitosan exhibits good cell adhesion but limited solubility. These limitations reduce their scalability and clinical translation, driving growing interest in hybrid bioinks.

#### 3.2.2. Synthetic Polymers

Synthetic polymers serve as customizable bioinks, allowing precise control over mechanical strength and physical characteristics. These polymers are fabricated using diverse technologies and platforms. Their composition, including biodegradability, can be finely regulated [76], overcoming the limitations of natural polymers. Key synthetic materials include polycaprolactone (PCL), polyethylene glycol (PEG), and polylactic acid (PLA). Most polymers are fabricated using 3D printing or electrospinning techniques [77]. PCL demonstrates exceptional structural stability and facilitates precise regulation of biocompatibility and degradation rates, making it a preferred material in brain tissue engineering [71]. PCL nanofibers generated using electrospinning effectively direct axon regeneration and enhance neuronal development [30]. The viscoelasticity of PEG-based hydrogels can be optimized by modifying crosslink density, facilitating the fabrication of diverse neuronal scaffolds [72].

#### 3.2.3. Hybrid Polymers

Hybrid polymer-based bioinks integrate the beneficial properties of both natural and synthetic polymers. They exhibit biological activity similar to that of natural polymers while offering mechanical strength comparable to synthetic polymers [72]. Modifying the physical properties of composite bioinks is relatively straightforward, and they effectively maintain cellular viability and structural integrity. For example, poly(3,4-ethylenedioxythiophene) (PEDOT) can be combined with polystyrene sulfonate (PSS) to create electrically conductive hydrogels [73]. Another method involves leveraging the ionic interactions between alginate and chitosan to enhance structural stability [74]. Hybrid bioink formulations regulate biodegradation rates and enhance the efficiency of cell adhesion and growth factor distribution. They effectively facilitate the fabrication of complex tissue architectures. The potential synergy among graphene, carbon nanotubes, and cellulose nanofibers has been investigated in several studies. This approach facilitates the development of effective, biocompatible bioinks for living organisms [75].

### 3.3. Key Design Considerations for Neural Bioprinting

#### 3.3.1. Biocompatibility and Cell Viability

In brain tissue engineering, bioinks and scaffolds must exhibit biocompatibility for effective 3D bioprinting applications. Maintaining cellular viability is essential for effective regeneration. Recent studies have explored the use of conductive hydrogels and nanomaterials. One effective strategy for achieving this involves integrating a small quantity of graphene oxide (GO) into gelatin methacryloyl (GelMA) hydrogels [78]. This modification enhances its mechanical strength and electrical conductivity. Cellular viability improves when exposed to UV light [78]. A scaffold composed of PCL/chitosan nanofibers integrated with gold nanoparticles has been developed. This material demonstrates minimal influence on neuronal development, attachment, or survival, indicating its biocompatibility and non-toxicity [79]. These findings highlight the critical role of bioink formulation and nanomaterial incorporation for supporting neuronal cell survival and function.

#### 3.3.2. Mechanical and Rheological Properties

Nerve tissue is classified as soft tissue with distinct mechanical properties. The mechanical strength and rheological behavior of scaffolds mimicking nerve tissue significantly affect cell adhesion, differentiation, and survival [80]. Recent studies have focused on regulating properties by combining conductive polymers with nanomaterials. A scaffold composed of polypyrrole (PPy) and silk fibroin (SF) is effective in promoting Schwann cell proliferation and neural regeneration through electrical stimulation [81,82]. Incorporating carboxylated graphene oxide into a PPy/poly-L-lactic acid (PLLA) composite enhances tensile strength, meeting the mechanical requirements of neural tissue [83]. These findings demonstrate that regulating mechanical and rheological properties improves scaffold performance in neural tissue engineering.

#### 3.3.3. Bioactivity and Electrical Conductivity

Electrical signal transmission is a fundamental function of nerve tissue. Therefore, scaffold electrical conductivity is an essential factor for neural cell growth and functional recovery. Recently, extensive research has focused on using conductive hydrogels and nanomaterials to enhance these properties [84]. For example, a conductive ink composed of polyaniline and polyethylene glycol diacrylate hydrogel exhibits semiconductor characteristics in scaffolds fabricated via 3D printing. These scaffolds maintain an average pore size of approximately 300 μm, offering a microenvironment suitable for nerve tissue regeneration [85]. Additionally, PEDOT:PSS-based hydrogels demonstrate high conductivity and stability. These hydrogels enhance neural cell adhesion and proliferation, demonstrating effectiveness in supporting axon formation and synaptic connections [86]. These findings highlight the importance of scaffold design considering both electrical conductivity and biological activity for successful neural tissue engineering applications.

## 4. Bioengineered Platforms for Peripheral Nervous System (PNS)

### 4.1. Biomaterial-Based Conduits for Peripheral Nerve Repair

**Figure 4:**
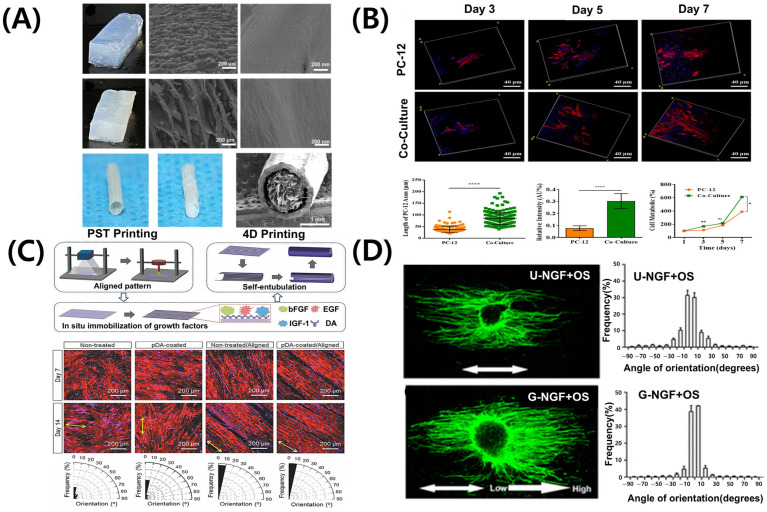
**Representative bioprinting strategies and scaffold designs for peripheral nerve regeneration.** (**A**) Hierarchical anisotropic silk-based nerve guidance conduit with aligned silk nanofiber fillers. (**B**) Schwann cell-laden GelMA hydrogel scaffold promoting neurite outgrowth. ns: no significant, * *p* < 0.05; ** *p* < 0.01, **** *p* < 0.0001. (**C**) 4D morphing scaffold using mussel-inspired adhesive chemistry for spatial growth factor patterning; black areas in the orientation histograms indicate angular ranges with no aligned fibers detected. (**D**) 3D printed scaffold with NGF gradient and oriented microchannels. Adapted with permission after [21,91,94,95].

The PNS exhibits inherent capacity for regeneration; however, severe or complex injuries often limit full recovery [87]. Consequently, tissue engineering approaches, particularly 3D bioprinting, have attracted significant attention. Nerve guidance conduits fabricated via 3D bioprinting offer structural support, promoting cell migration, alignment, and axon regeneration [88,89]. However, the success of these conduits depends on their architectural design and the selection of biomaterials that support cell viability, provide biochemical cues, and replicate the mechanical and degradation properties of native nerve tissue [90]. Alginate- and gelatin-based hydrogels exhibit good biocompatibility, improving cell survival and growth factor expression. Silk-based nerve conduits with hierarchical ECM-like structures effectively guide Schwann cells and neuronal axons (Figure 4A) [91]. Additionally, composites containing conductive polymers respond to electrical signals, enhancing cellular activity and accelerating nerve repair [92,93]. These material-based strategies offer significant advantages, combining structural support with physiological stimulation in nerve tissue engineering.

### 4.2. Schwann Cell-Based Scaffolds for Nerve Regeneration

Schwann cells are the principal glial cells of the PNS, essential for axonal regeneration and myelin sheath formation following nerve injury [96,97]. Advances in 3D bioprinting facilitate the direct incorporation of Schwann cells into biomaterials, creating optimal cell–scaffold composites for nerve regeneration. Figure 4B shows that GelMA hydrogels induce the attachment and alignment of Schwann cells [94]. Additionally, they enhance the secretion of nerve growth factors, such as NGF and BDNF [94], supporting axonal growth and differentiation. Scaffolds containing Schwann cells form axially aligned structures, guiding regenerating nerves in the correct direction. dECM-based bioinks of peripheral nerves have been developed in recent studies. These bioinks offer tissue-specific physiological cues [70,98,99], thereby promoting the functional maturation of both neurons and Schwann cells. 

### 4.3. Controlled Release Systems for Peripheral Nerve Regeneration

One key advantage of 3D bioprinting in peripheral nerve regeneration is its capacity to fabricate patient-specific structures. Scaffolds designed using CT or MRI imaging data demonstrate enhanced biocompatibility and superior therapeutic outcomes compared to conventional implants [100]. Figure 4C illustrates 3D-printed scaffolds used as drug delivery systems. These scaffolds are loaded with regeneration-promoting factors, such as NGF, GDNF, and VEGF, enabling their sustained release to support nerve regeneration [21,101]. Depending on the design of the scaffold, the release mechanisms may range from passive diffusion to stimuli-responsive systems activated by changes in pH, enzymatic activity, or external electrical stimulation [102]. These strategies allow spatiotemporal control over therapeutic delivery, thereby more closely mimicking the natural processes of nerve repair. The sequential or combined release of multiple growth factors, such as NGF for neurite extension followed by VEGF for angiogenesis, demonstrates synergistic effects in enhancing functional recovery [103]. Additionally, 3D printing technology enables the precise fabrication of multi-channel structures, promoting regeneration of different nerve types and supporting blood vessel growth [104]. Incorporating conductive or composite materials further stimulates axonal growth and enhances the re-establishment of functional neural connections [105].

### 4.4. Biophysical Gradients Platform for Axonal Guidance

Recent advances in microfabrication techniques enable the precise fabrication of microenvironments incorporating both chemical and mechanical gradients [106]. These gradient signals are crucial for peripheral nerve regeneration. Spatially controlled variations influence axonal growth, modulate Schwann cell activity, and enhance the alignment and functional maturation of regenerating nerve tissue. Growth factor-generated chemical gradients, such as NGF (Figure 4D), facilitate axonal extension and direct Schwann cell migration [95]. Consequently, Schwann cells secrete trophic molecules, including NGF and BDNF, further enhancing axonal growth [107,108]. Techniques such as microfluidic patterning and surface functionalization have been used to integrate these gradients into nerve conduits, thereby facilitating nerve regeneration. Peptide gradient patterns integrated into micropatterned scaffold surfaces replicate the natural ECM, enhancing functional recovery. Mechanical gradients, including variations in scaffold stiffness, influence cell adhesion, migration, and differentiation. For nerve regeneration, the optimal scaffold stiffness ranges from 0.9 kPa to 2.9 kPa [109]. These gradients affect both neurons and glial cells, guiding axonal growth and promoting the formation of functional connections [110]. Post-printing mechanical conditioning facilitates cell alignment and enhances scaffold integration in vivo [111]. Integrating chemical and mechanical gradients produces synergistic effects, enhancing regeneration outcomes. Hydrogels engineered with bioactive gradients and structural anisotropy facilitate neurite alignment and stimulate the release of growth factors [112]. Conductive polymers responsive to electrical stimulation integrate structural guidance with functional recovery, facilitating more effective nerve repair [113]. High-resolution bioprinting enables the creation of dynamic, biomimetic environments essential for successful peripheral nerve regeneration.

## 5. Bioengineered Platforms for Central Nervous System (CNS)

### 5.1. Biomaterial-Based Platforms for Central Nervous System Tissue Engineering

**Figure 5:**
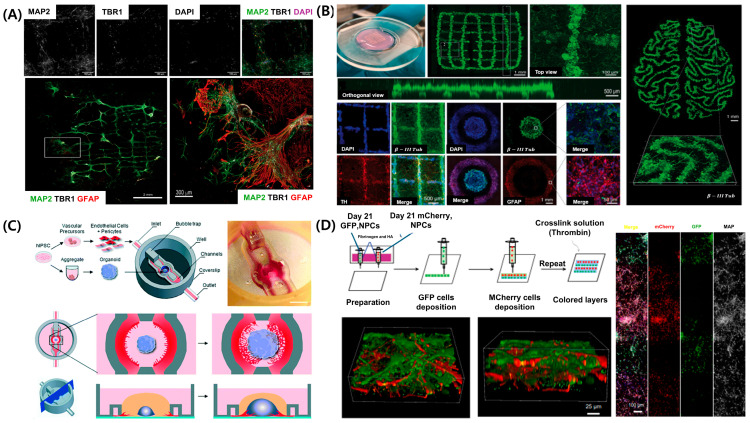
**Recent advancements in 3D bioprinted brain tissue models and CNS**-**on**-**chip platforms.** (**A**) Three-dimensional bioprinted cortical tissue model with regionally organized neuronal and astrocytic populations forming layered cortical architecture. (**B**) Brain organoid-based 3D printed scaffold supporting neural network formation with spatial patterning. (**C**) Microfluidic CNS-on-chip platform integrating vascularized brain organoid models. (**D**) Three-dimensional printed multilayered neural constructs with sequential deposition of fluorescently labeled neural precursor cells. Adapted with permission after [118,119,120,121].

The CNS, comprising the brain and spinal cord, serves as the primary control center for sensory information processing, motor coordination, and homeostatic regulation [114]. In contrast to the PNS, the CNS exhibits a highly complex structure, characterized by the presence of the BBB, intricate neural networks, and highly localized cellular architectures; however, its capacity for self-repair remains limited [115,116]. These characteristics pose significant challenges for investigating CNS disease and developing effective therapeutic strategies. The human brain comprises various cell types, including neurons, astrocytes, oligodendrocytes, and microglia, each organized into distinct regions [117]. Replicating this complexity in vitro requires advanced cell technologies capable of precise spatial arrangement of cells. Figure 5A illustrates that a 3D bioprinted construct, comprising induced pluripotent stem cell (iPSC)-derived neural cells, was developed using extrusion-based bioprinting. The bioprinted construct was validated using immunofluorescence staining for MAP2 (neuronal marker), TBR1 (deep-layer cortical neuron marker), and GFAP (astrocytic marker), confirming the successful formation of a layered, cortex-like architecture [118]. GelMA served as the primary bioink due to its high biocompatibility, tunable mechanical properties, and proven ability to support neuronal adhesion and differentiation. The GelMA hydrogel was photopolymerized to ensure structural stability, and its elastic modulus was adjusted to match that of brain tissue (~1 kPa). These biomaterial-based strategies support 3D structural integrity and offer a physiologically relevant environment that promotes neural cell maturation and the formation of functional synaptic network.

### 5.2. Layered Neural Constructs Using 3D Bioprinting

Traditional two-dimensional culture systems and animal models cannot sufficiently replicate the unique microenvironment of the CNS. To address these limitations, 3D bioprinting has emerged as a powerful technology for precisely depositing neural cells and biomaterials, creating models that more accurately mimic the cellular structure and functional properties of natural brain tissue (Figure 5B) [119]. For example, when human pluripotent stem cell-derived neural progenitor cells (NPCs) are patterned using extrusion-based bioprinting with GelMA-based bioinks [122], they exhibit spontaneous electrical activity and differentiate into mature neurons and astrocytes. The printed constructs self-organize into multilayered neural architectures resembling the cerebral cortex, further developing functional synaptic networks and demonstrating spontaneous calcium oscillations, indicating the formation of functional neural circuitry [123].

### 5.3. Vascularized Central Nervous System Models Developed with Microfluidic Platforms

Beyond layered tissue models, vascularization is essential for sustaining long-term cell viability and physiological function in CNS models. Figure 5C illustrates a microfluidic brain-on-a-chip platform used to model the vascular architecture of the brain [120]. This system integrates iPSC-derived brain microvascular endothelial cells, astrocytes, and pericytes into perfusable channels, mimicking both the structural and functional characteristics of the neurovascular unit (NVU). The model facilitates continuous perfusion, maintains oxygen and nutrient gradients, and supports long-term co-culture of neural cells with vascular networks. Functional performance was validated using transendothelial electrical resistance measurements and permeability assays, indicating the formation of a tight, selective BBB.

### 5.4. Developmental and Disease Models Using Bioprinted Central Nervous System Constructs

Brain development involves complex spatial and temporal signaling. Three-dimensional bioprinting models provide valuable platforms for investigating neural development and associated disorders, such as autism spectrum disorder, epilepsy, and intellectual disabilities [124,125,126]. Figure 5D illustrates that extrusion-based 3D bioprinting enables the spatially controlled deposition of human iPSC-derived NPCs and astrocyte precursors within bioinks, facilitating the reproduction of key developmental processes, including neuronal differentiation, synapse formation, cortical layering, and axon guidance [121]. The GelMA-based bioinks promote high cell viability and support long-term culture periods. Immunofluorescence staining confirms the formation of distinct neural layers, while calcium imaging demonstrates the development of functional synaptic activity. Additionally, iPSC-based personalized models facilitate the correlation of specific genotypes with phenotypic outcomes [118]. Bioprinted construct derived from NPCs and astrocyte precursors mature into functional neurons and astrocytes, exhibiting established synaptic connectivity and dynamic calcium signaling [56]. This approach enables the modeling of both developmental and pathological neural networks. Recent studies show that 3D CNS models replicate key pathological features of neurodegenerative diseases—for example, amyloid plaque formation in Alzheimer’s disease, dopamine neuron degeneration in Parkinson’s disease [127], and glioma progression [128]. These advances highlight the expanding range of applications, extending beyond neurodevelopmental research to include disease modeling and high-throughput drug screening.

### 5.5. Neural Cancer Models (e.g., Glioblastoma)

Glioblastoma multiforme (GBM) is an aggressive primary brain tumor characterized by high invasiveness and poor treatment response, largely due to its complex tumor microenvironment (TME) and the restrictive nature of the BBB [129]. Conventional models often fail to accurately mimic these pathological features [130]. Recent advances in 3D bioprinting have enabled the development of GBM models using ECM-mimetic bioinks combined with patient-derived GBM cells, endothelial cells, and supportive glial components [131]. These bioprinted constructs replicate key features of the TME, including vascular network formation and dynamic cell–cell interactions. Compared to conventional 2D cultures, they exhibit increased drug resistance and invasiveness. Furthermore, adjusting the ECM composition and stiffness affects angiogenic processes, including VEGF expression and blood vessel formation. Integrated NVU-GBM-on-a-chip platforms combining multi-nozzle bioprinting with microfluidic systems are currently in development, offering potential for high-throughput drug screening and personalized therapeutic testing [132]. However, challenges persist in the field, particularly the absence of immune components and the lack of standardized protocols [133]. Recent advances in 3D bioprinting and biomaterial engineering have facilitated the development of highly sophisticated CNS models, encompassing layered neural constructs, vascularized brain-on-a-chip systems, and disease-specific or TME platforms [23]. These bioengineered models closely replicate the structural, cellular, and functional characteristics of the human CNS, offering powerful tools for investigating neural development, modeling neurodegenerative and neoplastic disorders, and screening therapeutic candidates within physiologically relevant conditions.

## 6. Bioprinting Strategies for Central and Peripheral Nervous System Models

### 6.1. Comparative Analysis of Bioprinted Models of the Central and Peripheral Nervous Systems

The Three-dimensional bioprinting for neural tissue engineering should address the unique properties of the CNS and PNS, particularly regarding printing techniques, bioink composition, and design objectives. In CNS applications, bioprinting often focuses on replicating layered cortical architectures and interconnected synaptic networks. These constructs are typically fabricated using extrusion-based techniques with soft, neurocompatible hydrogels filled with neural progenitor and glial cells [66]. Achieving high spatial resolution, low shear stress, and long-term viability is crucial for neural tissue engineering. Furthermore, ongoing research focuses on implementing vascularization and synaptic functions by integrating microfluidic platforms that mimic the BBB [131]. In contrast, the PNS models primarily employ multi-material or coaxial printing to fabricate NGCs that facilitate chemical signaling and axon alignment. These models often use various bioinks (e.g., alginate, silk fibroin) with increasing interest in incorporating conductive elements to facilitate electrical signal transmission and support axonal growth. The primary priorities for tissue engineering are axon regeneration, Schwann cell support, and bone marrow formation. In contrast to the CNS, these processes have a limited effect on vascular integration. Both systems require careful optimization of printing parameters, such as extrusion pressure, nozzle diameter, and crosslinking conditions, to match the target tissue type. CNS models require precise replication of layered architecture and synaptic organization, while PNS structures emphasize axonal alignment and directional growth [134]. Furthermore, post-printing maturation strategies differ: CNS constructs typically undergo long-term neurotrophic culture to promote network development, while PNS constructs focus on early-stage guidance and repair processes [135].

### 6.2. Challenges and Opportunities in Bioprinting for Peripheral and Central Nervous Systems Regeneration

Despite recent progress, bioprinted disease models of both the CNS and PNS face significant limitations. These include challenges in accurately mimicking chronic disease progression, limited integration of immune system components, and the lack of standardized fabrication protocols. While bioprinting technologies establish spatial and biochemical gradients crucial for neural tissue modeling, current platforms often struggle to generate stable, high-resolution gradients with precise mechanical and biochemical control [136]. In PNS applications, significant technical challenges remain in achieving an optimal balance between bioink biocompatibility and mechanical strength [11]. Most hydrogel-based materials lack sufficient robustness for long-term in vivo stability. Advances in high-resolution printing, nozzle engineering, and post-processing techniques are necessary to replicate the complexity of neural architectures. Additionally, the development of bio-conductive, biodegradable materials is essential for enabling functional regeneration and seamless integration with neural devices. However, emerging innovations, including disease-specific bioinks [32], gradient-based drug delivery platforms, and organ-on-chip integrations, offer promising solutions [119]. These approaches will enhance the clinical relevance of neural models and expand the translational potential of 3D bioprinting in neuroregenerative medicine.

As summarized in Table 3, the central nervous system (CNS) and peripheral nervous system (PNS) models differ significantly in terms of bioprinting methods, cell types, bioink concentrations, tissue structure, functional verification, mechanical cues, and applications [66,87,88,89,90,91,92,131].

## 7. Future Directions and Challenges

### 7.1. Advancing Bioprinting Hardware for Gradient Control

Although 3D bioprinting has significantly advanced the reproduction of neural tissue structures, precise control over the mechanical, biochemical, and electrical gradients crucial for neural development and regeneration remains a major challenge. Functional graded materials are crucial for replicating the heterogeneous microenvironment of neural tissue, but current bioprinting systems cannot generate such gradients continuously and accurately [137]. Traditional nozzle systems are primarily designed for uniform extrusion, limiting dynamic control over bioink composition and rheological properties during printing [138]. This limitation hinders the reproduction of regional microenvironments crucial for neural differentiation, synapse formation, and axonal pathfinding. To address these limitations, innovative nozzle designs have been proposed. Multi-material bioprinting platforms incorporating coaxial, triaxial, or microfluidic-integrated nozzles enable spatially controlled co-extrusion and mixing of multiple bioinks, facilitating continuous or discrete gradient formation in situ [139]. This approach supports the fabrication of complex tissue interfaces and physiologically relevant segmented neural structures. However, achieving precise, reproducible, and stable gradient patterns remains technically challenging. Recent advances in closed-loop control systems incorporating machine learning (ML) algorithms offer promising solutions [140]. These systems dynamically regulate key printing parameters such as pressure, flow rate, and temperature in real time to enhance gradient fidelity and consistency. However, further efforts are required to standardize these advanced hardware systems and to validate their scalability for translational and clinical applications. Moreover, translating 3D bioprinted neural constructs from bench to bedside remains a formidable challenge. Current barriers include limited batch-to-batch reproducibility, the absence of regulatory frameworks specific to bioprinted neural tissues, and difficulties in achieving stable functional integration with host tissues. Additionally, issues related to immune compatibility, long-term safety, and large-scale manufacturing capacity must be resolved to advance clinical adoption.

### 7.2. Advancements in Bioink Development

The performance and functionality of bioinks are crucial for successful bioprinting. Conventional hydrogel bioinks, including GelMA, alginate, and collagen, are widely used for their biocompatibility and shear-thinning properties. However, balancing mechanical strength, cellular viability, and printability remains challenging. Soft hydrogels lack sufficient mechanical integrity, whereas rigid materials compromise cell health. To address these limitations, hybrid and composite bioinks have been developed. dECM bioinks provide tissue-specific environments that promote cell differentiation and maturation [141]. Conductive bioinks incorporating materials such as polypyrrole or graphene are used in neural and cardiac tissue engineering [142]. These bioinks respond to electrical stimuli, facilitating signal transmission and functional restoration. Furthermore, Ongoing research is also focused on developing stimulus-responsive bioinks that react to temperature, pH, or light [143,144]. Recent efforts have emphasized the establishment of standardized protocols for bioink evaluation, including rheological characterization, biocompatibility analysis, and degradation assessment [145].

### 7.3. Integration of Bioprinting with Complementary Technologies

The advancement in 3D bioprinting increasingly emphasizes its integration with complementary technologies, including artificial intelligence (AI), ML, organ-on-a-chip (OoC) [146], and microchip platforms. Artificial neural networks in AI and ML can monitor and assess printing status. Deep learning models enhance scaffold design optimization, reducing printing errors and accelerating experimental timelines [147,148]. The integration of 3D bioprinting with OoC and microfluidic technologies is currently advancing. These technologies simulate fluid flow, shear stress, and tissue-specific microenvironments [149]. Incorporating bioprinted tissues into chip-based systems sustains organ viability, maturation, and tissue functionality [150,151]. This hybrid technology facilitates the development of models for multi-organ interactions, personalized medicine, and high-throughput drug screening. Microfluidic chips can be coupled with Interstitial spaces or bioprinted tumor tissues. This system has been applied to investigate metabolism, cancer progression, and immune responses. Integrated biosensors for pH, oxygen, and cytokine detection enhance real-time monitoring [152,153]. The integration of AI and microfluidic engineering is gaining considerable attention. AI-driven control systems regulate fluid dynamics based on cellular conditions to accurately replicate biological environments. Certain systems employ machine learning-driven feedback loops to improve and adjust manufacturing processes [154,155]. Persistent challenges include ensuring bioink–substrate compatibility, maintaining long-term perfusion, and managing data in AI-integrated systems. Ongoing interdisciplinary collaboration is crucial to overcoming these barriers. 

## 8. Conclusions

In summary, 3D bioprinting technology offers significant potential for neural tissue engineering by enabling precise replication of intricate cellular structures and circuit formation in the CNS and PNS. This approach addresses key limitations of traditional two-dimensional cultures and animal models. This review highlighted the significance of bioink properties, such as rheology, biocompatibility, bioactivity, and conductivity, in ensuring structural stability and cellular function. Furthermore, precise control of scaffold microstructure and mechanical properties, combined with the use of bifunctional materials, enhances the modulation of neuronal and Schwann cell activity. Incorporating patient-derived iPSCs for personalized disease modeling, alongside brain organoids and spatiotemporal regulation of growth factors and electrical stimulation, offers significant potential for advancing drug screening and regenerative therapies. Future research should prioritize sophisticated bioprinting techniques that enable multicellular patterning, vascularization, and electrophysiological integration. This focus will support the development of advanced neural tissue platforms for functional reconstruction and clinical application.

## Figures and Tables

**Table 1:** Comparison of bioprinting techniques in neural tissue engineering.

Parameter	Extrusion Bioprinting	Inkjet Bioprinting	EHD Bioprinting	Laser-Assisted Bioprinting	Stereolithography	Two-Photon Polymerization
Speed	Slow	Fast	Medium–High	Medium	Fast	Slow
Cost	Moderate	Low	High	High	Low	Very High
Cell Viability	85–95%	80–95%	>90%	<85%	>85%	>80%
Cell Density	High	Low–Moderate	Very High	Medium	Medium	Medium
Resolution	100–500 µm	100–300 µm	50–100 nm to 10 µm	20–100 µm	10–50 μm	0.1–1 μm
Viscosity Range	30–6 × 10^7^ mPa·s	<10 mPa·s	1–1000 mPa·s (wide range)	1–300 mPa·s	No limitation	Mo limitation
Material Type	Hydrogels (e.g., GelMA, alginate), ECM-based materials	Low viscosity inks (e.g., modified hydrogels)	Conductive or ECM-based hydrogels	ECM-based, photosensitive materials	Photocurable polymers (e.g., PEGDA, GelMA)	Photosensitive resins (e.g., acrylates, GelMA derivatives)
Bioink Flexibility	High (multi-material, high cell load)	Moderate	High (nanoscale jetting of bioinks)	Moderate	Moderate	Low
Application	General tissue models, nerve guidance conduits	Patterning, high-throughput screening	Fine patterning, micro/nanoscale architecture	High precision scaffolds, neuroanatomical layering	Vascular and neural constructs with precise geometry	Ultra–high-resolution microstructures
Limitations	Low resolution, nozzle clogging	Droplet formation constraints	High voltage risk, complex setup	Expensive setup, limited cell survival	Limited cell survival, limiting of bioinks	Very slow printing, expensive setup
Reference	[45,46,47,48]	[49,50]	[51,52,53]	[54,55,56]	[57]	[58]

**Table 2:** Properties and applications of different bioinks used in neural tissue engineering.

Category	Material	Key Properties	Applications	References
Natural Polymers	Collagen	Enhances cell viability and proliferation	Peripheral nerve conduits	[64,65]
	HA	Promotes neurite outgrowth and neural differentiation	PNS and CNS therapeutic scaffolds	[66]
	Chitosan	Supports cell adhesion and neural regeneration	Scaffold for PNS and CNS regeneration	[67]
	Alginate	Facilitates scaffold development for nerve regeneration	Blended scaffolds for nerve regeneration	[68]
	dECM	Enables cell-specific adhesion and functional differentiation	Bioink for CNS and PNS bioprinting	[69,70]
Synthetic Polymers	PLA	Biodegradable; modifiable for structural requirements	3D printing and electrospinning	[71,72]
	PCL	High structural stability; tunable degradation rate	Electrospun fibers for axon guidance	[71]
	PEG	Tunable viscoelasticity, suitable for scaffold design	Hydrogels for tunable scaffold architecture	[72]
Hybrid Polymers	PEDOT:PSS	Conductive hydrogel with favorable electrical properties	Neural scaffolds requiring electrical stimulation	[73]
	Alginate–Chitosan	Enhanced structural stability via ionic interaction	Hybrid scaffolds for neural regeneration	[74]
	Graphene/Carbon Nanotubes/ Cellulose Nanofibers	Enhanced electrical conductivity and bioactivity	Advanced functional bioinks for complex tissue fabrication	[75]

**Table 3:** Comparative Analysis of the CNS and PNS.

Category	CNS Models	PNS Models
Bioprinting Method	Slow	Fast
Cell Types	NPCs, astrocytes, neurons	PC-12, SH-SY5Y, Schwann cells, neurons
Bioink Concentration	85–95%	80–95%
Tissue Architecture	High	Low–Moderate
Functional Validation	100–500 µm	100–300 µm
Mechanical Cues	30–6 × 10^7^ mPa·s	<10 mPa·s
Applications	Hydrogels (e.g., GelMA, alginate), ECM	Low-viscosity inks (e.g., modified hydrogels)
Vascularization	High (multi-material, high cell load)	Moderate
Category	General tissue models, nerve guides	Patterning, high-throughput screening
Reference	[66,131]	[87,88,89,90,91,92]

## Data Availability

The data presented in this study are available on request from the corresponding author.

## Abbreviations

3D	Three-dimensional
4D	Four-dimensional (time-responsive) bioprinting
AI	Artificial intelligence
BBB	Blood–brain barrier
CNS	Central nervous system
DAPI	4′,6-diamidino-2-phenylindole
dECM	Decellularized extracellular matrix
DLP	Digital light processing
ECM	Extracellular matrix
EHD	Electrohydrodynamic
GelMA	Gelatin methacryloyl
GFP	Green fluorescent protein
HA	Hyaluronic acid
MAP2	Microtubule-associated protein 2
NGF	Nerve growth factor
NPCs	Neural progenitor cells
NTE	Neural tissue engineering
PCL	Polycaprolactone
PEDOT:PSS	Poly(3,4-ethylenedioxythiophene):poly(styrenesulfonate)
PEG	Polyethylene glycol
PLA	Polylactic acid
PNS	Peripheral nervous system
PST	Phase separation technique
TBR1	T-box brain transcription factor 1
VEGF	Vascular endothelial growth factor
